# Sirt2 in the Spinal Cord Regulates Chronic Neuropathic Pain Through Nrf2-Mediated Oxidative Stress Pathway in Rats

**DOI:** 10.3389/fphar.2021.646477

**Published:** 2021-04-09

**Authors:** Mengnan Zhao, Xiaojiao Zhang, Xueshu Tao, Bohan Zhang, Cong Sun, Pinying Wang, Tao Song

**Affiliations:** Department of Pain Medicine, The First Hospital of China Medical University, Shenyang, China

**Keywords:** SIRT2, NRF2 activity, oxidative stress, the spinal cord, chronic neuropathic pain

## Abstract

Reduction in Nrf2-mediated antioxidant response in the central nervous system plays an important role in the development and maintenance of neuropathic pain (NP). However, the mechanisms regulating Nrf2 activity in NP remain unclear. A recent *in vitro* study revealed that Sirt2, a member of the sirtuin family of proteins, affects antioxidant capacity by modulating Nrf2 activity. Here we examined whether central Sirt2 regulates NP through Nrf2-mediated oxidative stress pathway. In a rat model of spared nerve injury (SNI)-induced NP, mechanical allodynia and thermal hyperalgesia were observed on day 1 and up to day 14 post-SNI. The expression of Sirt2, Nrf2 and its target gene NQO1 in the spinal cord in SNI rats, compared with sham rats, was significantly decreased from day 7 and remained lower until the end of the experiment (day 14). The mechanical allodynia and thermal hyperalgesia in SNI rats were ameliorated by intrathecal injection of Nrf2 agonist tBHQ, which normalized expression of Nrf2 and NQO1 and reversed SNI-induced decrease in antioxidant enzyme superoxide dismutase (SOD) and increase in oxidative stress marker 8-hydroxy-2′-deoxyguanosine (8-OHdG) in the spinal cord. Moreover, intrathecal injection of a recombinant adenovirus expressing Sirt2 (Ad-Sirt2) that upregulated expression of Sirt2, restored expression of Nrf2 and NQO1 and attenuated oxidative stress in the spinal cord, leading to improvement of thermal hyperalgesia and mechanical allodynia in SNI rats. These findings suggest that peripheral nerve injury downregulates Sirt2 expression in the spinal cord, which inhibits Nrf2 activity, leading to increased oxidative stress and the development of chronic NP.

## Introduction

Neuropathic pain (NP), which is redefined as a “pain caused by lesion or disease of the somatosensory system,” is an underestimated socioeconomic health problem affecting millions of people worldwide (Carrasco et al., 2018). A systematic review of epidemiological studies has estimated that the prevalence of NP is 6.9–10% (St. John Smith, 2018). The clinical symptoms of NP are different, including spontaneous pain, hyperalgesia, allodynia and paresthesia (Nickel et al., 2012), and the most commonly prescribed analgesics generally are less effective for NP. NP can become a chronic and hardly bearable condition, leading to increased episodes of depression and suicide in some case (Torrance et al., 2013). A better understanding of the molecular mechanism underlying NP may lead to improvements in pain relief and quality of life in patients with NP.

Oxidative stress has been suggested to play an important role in the development and maintenance of neuropathic pain (Carrasco et al., 2018; Shim et al., 2019). Excessive reactive oxygen species (ROS) has a deleterious effect on organelles, antioxidant defenses and other biomolecules, leading to mitochondrial dysfunction, glial activation and inflammatory response. This adverse environment is ultimately responsible for the typical painful symptoms of NP (Carrasco et al., 2018). Nuclear factor erythroid derived-2-related factor 2 (Nrf2) is a transcription factor and master regulator of many antioxidant/detoxification genes. Nrf2 pathway has been considered as a critical cellular defense mechanism against oxidative stress (Lee and Johnson, 2004; Kaspar et al., 2009; Catanzaro et al., 2017). Accumulating evidence shows that Nrf2 pathway is involved in the pathogenesis of NP (Zhou et al., 2020; Pol, 2021), but the mechanism that mediates Nrf2 pathway in NP is unclear.

The sirtuins (sirts) are a family of nicotinamide adenine dinucleotide (NAD+)-dependent histone deacetylases (HDACs) that play important roles in many cellular functions, including histone deacetylation, protein acylation, and deacetylation (Singh et al., 2018). In addition, sirtuins have protective properties, antioxidant-promoting actions and ROS-suppressive effects in mammalian cells (Singh et al., 2018). For example, overexpression of Sirt2, a member of the sirtuin family, decreases levels of ROS and increases the expression of antioxidant enzymes such as MnSOD, catalase, and glutathione peroxidase in the cells treated with lipopolysaccharides or hydrogen peroxide (Kim et al., 2013). A recent study showed that inhibition of Sirt2 with its inhibitor AGK2 attenuated the (NAD+) -induced increases in Nrf2 mRNA expression and nuclear Nrf2 levels, which were accompanied by reduced antioxidant capacity in PC12 cells (Zhang et al., 2019). Moreover, Sirt2 has ability to regulate nuclear Nrf2 levels and its downstream antioxidant gene expression by modulating AKT phosphorylation (Cao et al., 2016). In the present study, we examined whether Sirt2 regulates NP through Nrf2-mediated oxidative stress. For this purpose, we used a spared nerve injury (SNI)-induced NP model, which mimics human NP related to peripheral nerve injury.

## Materials and Methods

### Animals

Adult male Sprague-Dawley rats weighing 180–220 g were purchased from Liaoning Changsheng Biological Center, Shenyang, China. The rats were housed in separated cages at 23–25°C and 50–60% humidity under a 12/12-h light/dark cycle with free access to water and food. The animal experiments were performed according to the Guiding Principles for Research Involving Animal and Human Beings, and the experimental procedures were approved by the Animal Care and Use Committee of China Medical University (IACUC no. 2019028).

### Experimental Protocols

#### Protocol I

**FIGURE 1:**
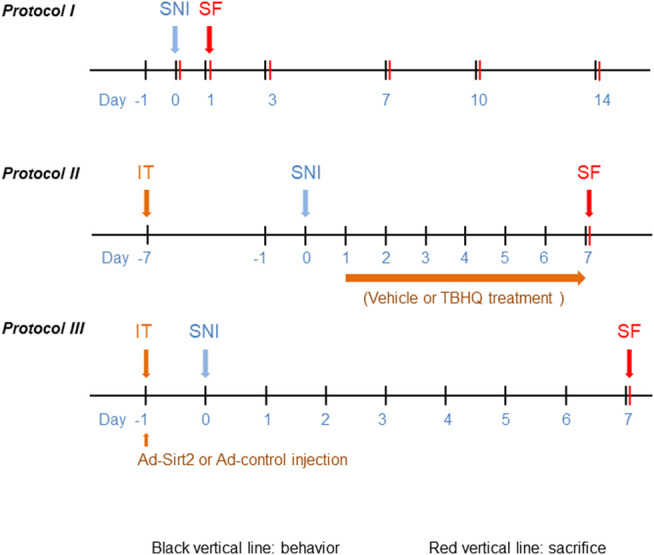
Schematic illustration of the experimental protocols. SNI: spared nerve injury; IT: implantation of lumbar intrathecal catheter; SF: sacrifice.

To examine the time course of changes in pain behaviors and expression of Nrf2, Sirt2 and NQO1 in the spinal cord, rats were assigned to sham (n = 5) and SNI (n = 30) groups. The SNI group underwent SNI surgery, while sham group received sham operation. Paw withdrawal threshold (PWT) to mechanical stimulation and paw withdrawal latency (PWL) to thermal stimulation were assessed at the ipsilateral hind paws 24 h prior to SNI and day 1, 3, 7, 10 and 14 post SNI. Five SNI rats were sacrificed after PWT and PWL measurement at each time point and sham rats were sacrificed at final time point. The protein was extracted from L4-6 spinal cord of injured side for molecular studies (Figure 1A).

#### Protocol II

To examine the role of Nrf2 in the spinal cord in regulation of NP, rats were divided into 5 experimental groups (n = 4 for each group): sham; SNI without treatment; SNI treated with DMSO (vehicle); SNI treated with Nrf2 agonist tBHQ at 1 μM; and SNI treated with tBHQ at 10 μM. Lumbar intrathecal catheter implantation was conducted 7 days prior to SNI. DMSO and tBHQ (dissolved in 10 μL DMSO) was intrathecally injected once a day for 7 days, starting from day 1 after SNI. PWT and PWL were measured daily and animals were sacrificed at the end of the protocol to collect spinal cord tissues for molecular studies. Additional SNI rats treated with DMSO and tBHQ at 10 μM (n = 3 for each group) were used for immunofluorescent study at the end of the protocol (Figure 1B). The doses of tBHQ used in this study for intrathecal injection were based on our preliminary experiment showing that intrathecal injection of tBHQ at 1 μM significantly increased spinal Nrf2 expression and that intrathecal injection of tBHQ at 10 μM induced a greater increase in spinal Nrf2 expression in rats.

#### Protocol III

To determine the role of Sirt2 in regulation of Nrf2 pathway, rats were divided into 4 experimental groups (n = 5 for each group): sham; SNI without treatment; SNI treated with a recombinant adenovirus expressing control and GFP (Ad-control); and SNI treated with a recombinant adenovirus expressing Sirt2 and GFP (Ad-Sirt2, 1 × 10^8^ PFU in 10 μL saline). Ad-control and Ad-Sirt2 were intrathecally injected 24 hours prior to SNI. PWT and PWL were measured daily and animals were sacrificed at the end of the protocol to collect spinal cord tissues for molecular studies. Additional SNI rats treated with Ad-control and Ad-Sirt2 (n = 3 for each group) were used for immunofluorescent study at the end of the protocol (Figure 1C).

### Induction of Neuropathic Pain Model

The rat model of neuropathic pain was induced by unilateral SNI as previously described (Decosterd and Woolf, 2000; Guo et al., 2019). Briefly, rats were anesthetized by inhalation of 3% isoflurane, and a section was made directly through the biceps femoris muscle to expose the sciatic nerve and its three terminal branches: the common peroneal, tibial and sural nerves. The common peroneal and the tibial nerves were ligated tightly with 5.0 silk and transected distal to the ligation. Approximately 2–3 mm of the distal nerve stump was then excised. Muscle layers were closed using 4-0 chromic gut, and the skin incision was closed with wound staples. In sham surgery, the sciatic nerves were exposed but not ligated. Rats with sham surgery were used as control.

### Implantation of Lumbar Intrathecal Catheter

Lumbar intrathecal catheter implantation was conducted as previously described (Størkson et al., 1996; Guo et al., 2019). Briefly, under 3% isoflurane anesthesia, an incision lateral to the midline was made and the polyethylene catheter was inserted into the subarachnoid space. The correct intrathecal localization was confirmed by a tail-flicking action and hind limb paralysis after administration of 2% lidocaine (10 µL) through the catheter in wakened animals.

### Western Blot Analysis

Spinal cord (L4-6) was homogenized in ice-cold lysis buffer (Sigma-Aldrich, St. Louis, MO, United States) containing protease inhibitor cocktail. Supernatants were collected by centrifugation at 12,000 × g for 20 min at 4°C. The nuclear protein from spinal cord was prepared using a NE-PER Nuclear and Cytoplasmic Extraction Reagents (Thermo Fisher Scientific, Waltham, MA, United States) according to the manufacturer’s instructions. Protein concentrations were determined with the BCA protein assay (Thermo Fisher Scientific, Waltham, MA, United States). Equal amounts of protein were separated by 8% SDS-electrophoresis and transferred onto polyvinylidene difluoride membranes. The membranes were placed in blocking buffer (5% milk in Tris-buffered saline with Tween-20) for one hour and then incubated over night at 4°C with primary antibodies to Sirt2 (1:500, Abcam, Cambridge, United Kingdom), Nrf2 (1:1,000, Abcam, Cambridge, United Kingdom), NQO1 (1:10,000, Abcam, Cambridge, United Kingdom), lamin B1 (1:1,000, Cell Signal Technology, MA, United Kingdom) and β-actin (1:1,000, Cell Signal Technology, Beverly, MA, United States). After incubation with horseradish peroxidase-conjugated secondary antibodies (1:10,000, Santa Cruz Biotechnology, Santa Cruz, CA, United States) for 1 h at room temperature, the labeled proteins were visualized by enhanced chemiluminescence detection system (GE Healthcare, Waukesha, WI, United States) and analyzed with ImageJ software (NIH, Bethesda, Maryland, United States). Results were normalized to β-actin or lamin B.

### Measurements of Antioxidants and Oxidative Products

Spinal cord samples were collected and lyzed with RIPA buffer. The enzymatic activity of superoxide dismutase (SOD) was measured using the T-SOD assay kit (Jiancheng Bioengineering Institute, Nanjing, China) and levels of 8-hydroxy-2′-deoxyguanosine (8-OHdG) were determined using ELISA kits (Thermo Scientific, Rockford, IL, United States) according to the manufacturer’s instructions.

### Assessments of Mechanical Allodynia and Thermal Hyperalgesia

Mechanical allodynia was assessed by measuring the paw withdrawal threshold (PWT) in response to the stimulation of Von Frey filaments, as previously described (Chaplan et al., 1994; Guo et al., 2019). Briefly, rats were acclimatized in a plastic cage with a mesh bottom for 20 min prior to testing. PWT was assessed using a dynamic plantar esthesiometer (Ugo Basile, 37450, Italy), which consists of a force transduction fitted with a 0.5-mm diameter polypropylene rigid tip. A probe was applied perpendicularly to the mid-plantar surface of the hind paw with an increasing pressure. The cutoff pressure was set to be 50 g and the force that induced the withdrawal response was automatically recorded by the esthesiometer. For each animal, at least three measurements were performed with an interval of 5 min for the stimulation of each hind paw. Thermal hyperalgesia was determined by measuring the paw withdrawal latencies (PWL) in response to heat plate test. A hot plate (Ugo Basile Srl 7280, Gemonio, Italy) with a pre-set plate temperature of 52.5 °C as recommended for rats (Hestehave et al., 2019) was used. As soon as the rat was placed onto the hot plate, the time between placement and licking, shaking or stepping of the hindpaws was recorded (Bannon and Malmberg, 2007). A cut-off-time was set at 30s to avoid tissue damage.

### Immunofluorescent Study

The immunofluorescent study was performed as previously described (Wang et al., 2014), Briefly, rats were transcardially perfused with saline containing heparin (1 unit/ml) followed by 4% paraformaldehyde in 0.1 M PBS. Spinal cords were removed and fixed overnight in 4% paraformaldehyde at 4°C and cryoprotected with 30% sucrose in 0.1 M PBS for 2 days. The spinal cords were sliced into 18-µm coronal sections. After being blocked with 5% goat serum in 0.3% Triton for 1 h at room temperature, the sections were incubated with primary to Sirt2 (1:200, Abcam, Cambridge, United Kingdom or Nrf2 (1:200, Abcam, Cambridge, United Kingdom) overnight at 4 °C, followed by Alex Fluor 568 second antibody (1:200, Thermo Fisher Scientific, Waltham, MA, United States). DAPI was used for nuclear staining. Images were captured using a confocal laser-scanning microscope (Zeiss LSM 510, Carl Zeiss, Inc.).

### Statistical Analysis

All data are presented as the mean ± SE. Statistical analyses were performed using GraphPad Prism 7 (GraphPad Software, Inc.). The differences between groups were analyzed by a one-way or two-way analysis of variance (ANOVA) followed by Bonferroni post hoc tests for multiple comparisons. Statistical significance was reached with P values below 0.05.

## Results

### Time Course of Changes in Pain Behaviors and Expression of Nrf2 and its Downstream Target in the Spinal Cord Following SNI

**FIGURE 2:**
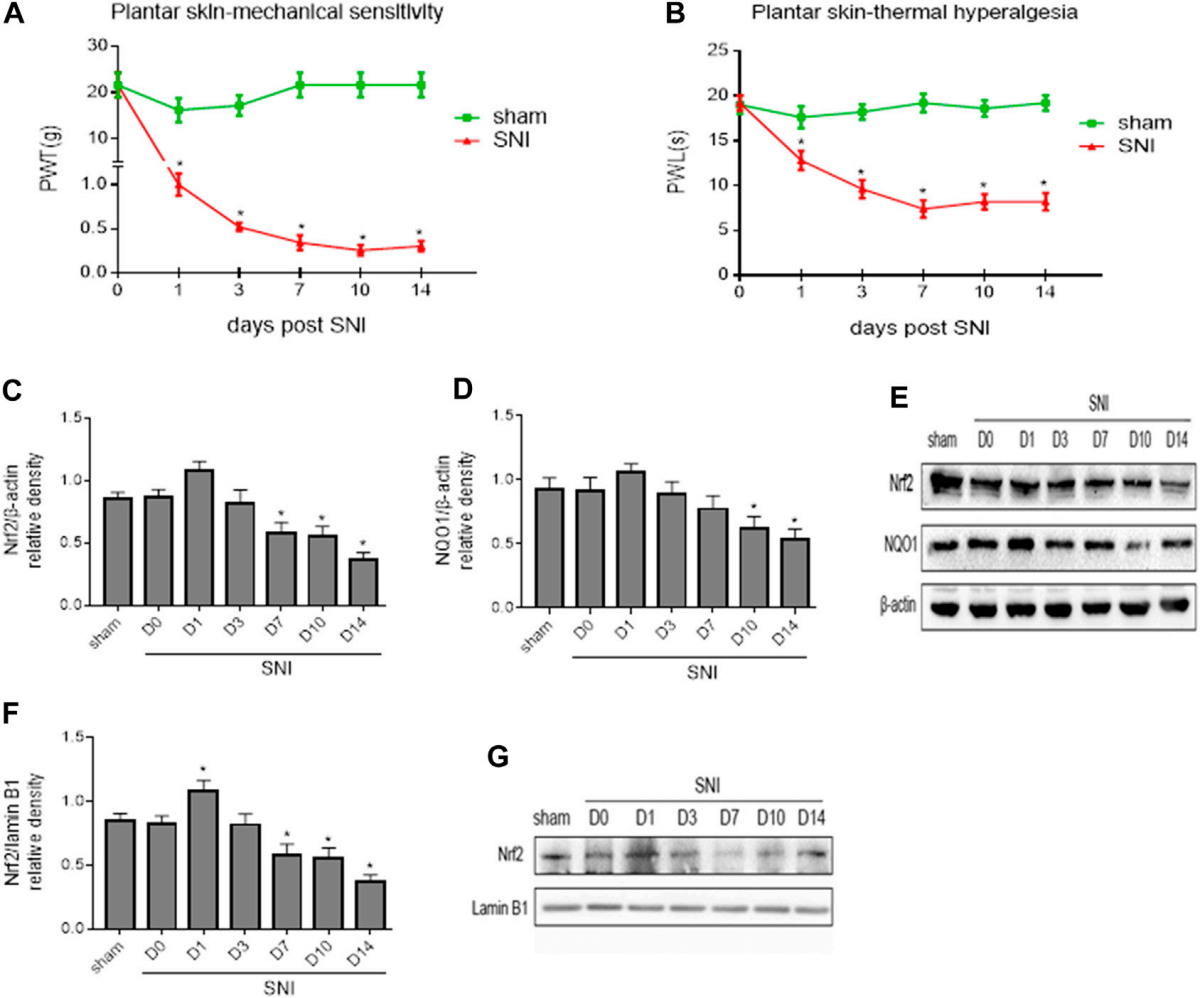
**(A,B)**: Paw withdrawal threshold (PWT) to mechanical stimulation and paw withdrawal latency (PWL) to thermal stimulation before (day 0) and 1, 3, 7, 10 and 14 days after spared nerve injury (SNI) or sham operation. **(C–E)**: The time course of changes in expression of Nrf2 and its downstream target NQO1 in the whole tissue lysates of the spinal cord in SNI rats. **(F,G)**: The time course of changes in expression of Nrf2 in the nuclear fractions of the spinal cord in SNI rats. Sham rats served as control. Values are expressed as mean ± SE (n = 5 for each group). *p < 0.05 vs sham or baseline (day 0).

Baseline measures of mechanical allodynia and thermal hyperalgesia were recorded 24 h prior to SNI to determine preinjury thresholds. NP behaviors were examined on 1, 3, 7, 10 and 14 days following SNI. As shown in Figure 2, there were no differences in mechanical allodynia (Figure 2A) and thermal hyperalgesia (Figure 2B) between groups at baseline. SNI induced significant mechanical allodynia as indicated by decreased PWT and thermal hyperalgesia as evidenced by reduced PWL within 1 day and lasting up to 14 days compared to sham controls and baseline. The maximal mechanical allodynia and thermal hyperalgesia in SNI group were observed at day 10 and day 7, respectively.

To explore the potential role of Nrf2 pathway in regulation of NP, we first examined the expression of Nrf2 and NQO1, a downstream target of Nrf2 that plays a protective role in various cells against oxidative stress, in the spinal cord of SNI rats. Western blot analysis revealed that the expression of Nrf2 (Figures 2C,E) and NQO1 (Figures 2D,E) in the whole tissue lysates of SNI group tended to be higher at day 1, but gradually decreased from day 7 following SNI, compared with baseline or sham group. Expression of Nrf2 in the nuclear fractions (Figures 2F,G) in SNI group was significantly increased at day 1, but then started to gradually decrease in the following day. Significant reduction in expression of Nrf2 in the nuclear fractions of SNI group was observed from day 7 as compared to baseline or sham group.

### Activation of Nrf2 Pathway in the Spinal Cord Ameliorates NP

**FIGURE 3:**
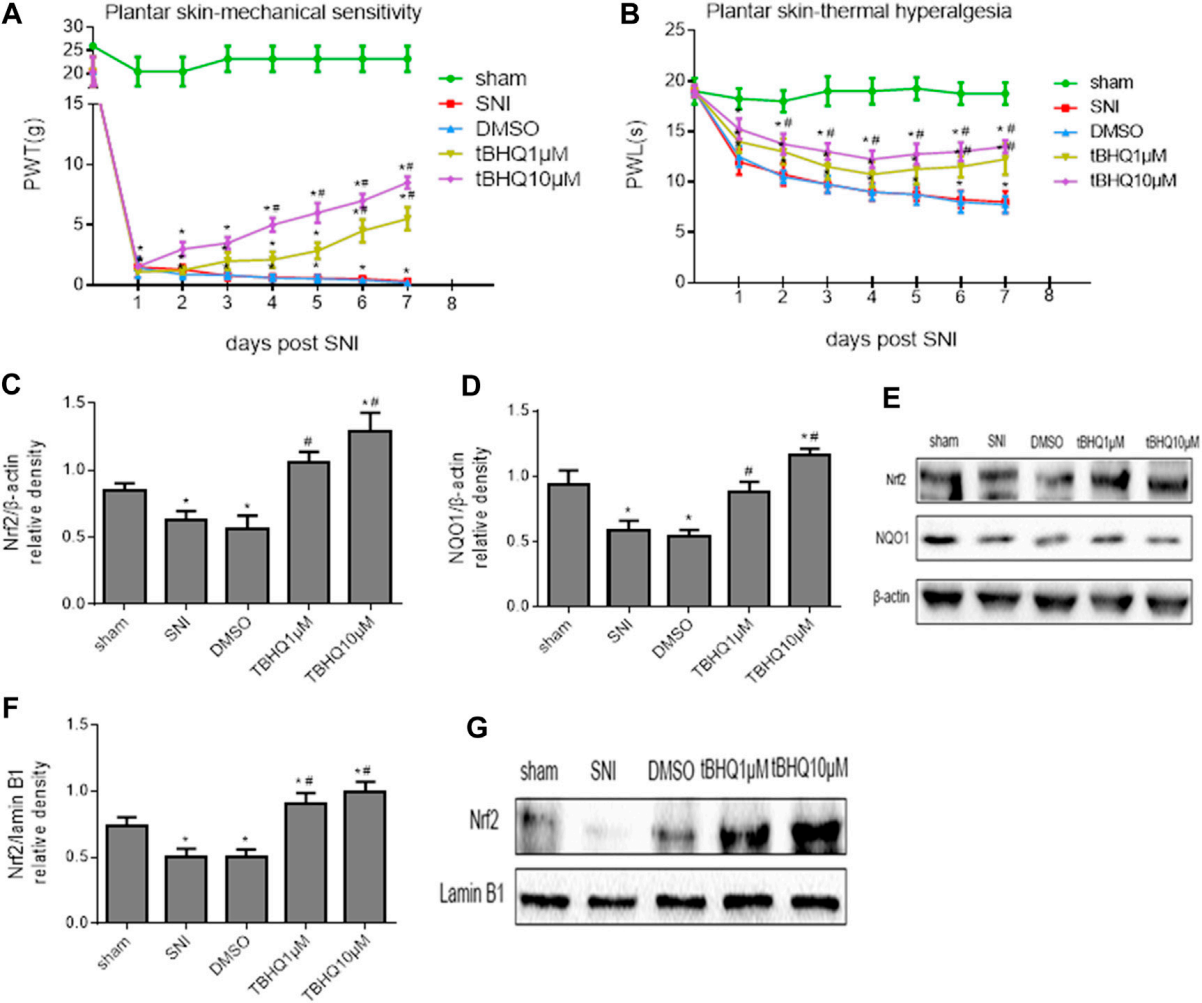
**(A,B)**: Effects of intrathecal injection of DMSO (vehicle) or different doses of Nrf2 agonist tBHQ on mechanical allodynia and thermal hyperalgesia in SNI rats. **(C–E)**: Effects of intrathecal injection of DMSO or different doses of Nrf2 agonist tBHQ on expression of Nrf2 and its downstream target NQO1 in the whole tissue lysates of the spinal cord in SNI rats. **(F,G)**: Effects of intrathecal injection of DMSO and different doses of Nrf2 agonist tBHQ on expression of Nrf2 in the nuclear fractions of the spinal cord in SNI rats. Sham rats without treatment served as control. Values are expressed as mean ± SE (n = 4 for each group). *p < 0.05 vs sham or baseline (day 0); ^#^ *p* < 0.05 vs SNI or DMSO.

To examine whether activation of Nrf2 pathway in the spinal cord of SNI rats would ameliorate NP, SNI rats were treated with intrathecal injection of DMSO (vehicle) or Nrf2 agonist tBHQ at different doses. The mechanical allodynia (Figure 3A) and thermal hyperalgesia (Figure 3B) in SNI rats, compared with SNI rats without treatment, were attenuated by intrathecal tBHQ at both doses from day 4 for PWT and day 2 for PWL, respectively. Intrathecal injection of DMSO had no effects on mechanical allodynia and thermal hyperalgesia in SNI rats.

Western blots confirmed that expression of Nrf2 (Figures 3C,E) and NQO1 (Figures 3D,E) in the whole tissue lysates and expression of Nrf2 in the nuclear fractions (Figures 3F,G) in SNI rats without treatment were significantly decreased when compared with sham rats. Intrathecal tBHQ at either dose, but not DMSO, increased expression of Nrf2 and NQO1 in the whole tissue lysates and expression of Nrf2 in the nuclear fractions.

**FIGURE 4:**
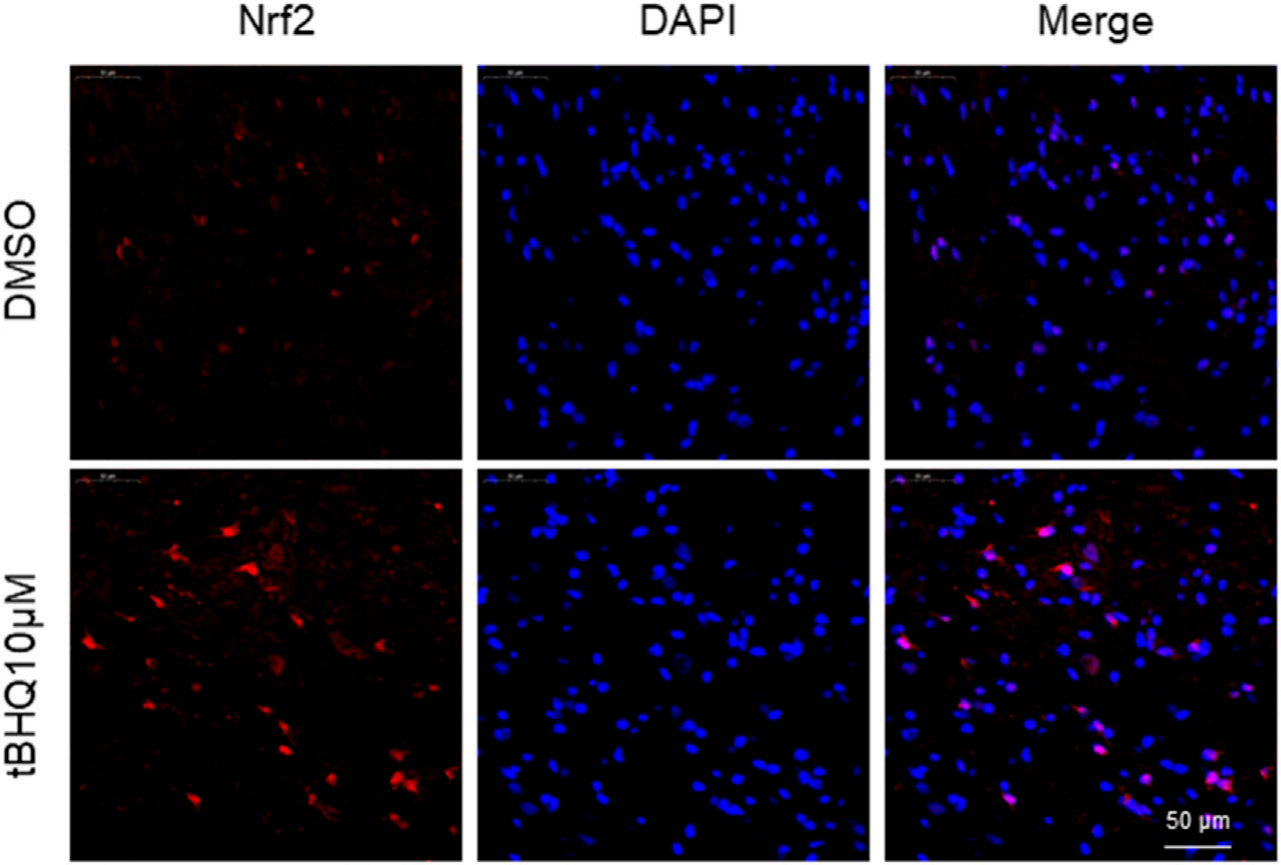
Representative confocal images showing Nrf2 immunoreactivity (red) in the spinal cord in SNI rats treated with DMSO (vehicle) or Nrf2 agonist tBHQ at 10 μM. The nuclear staining by DAPI is shown in blue.

Immunofluorescent study showed that SNI rats treated with intrathecal tBHQ at a dose of 10 μM exhibited abundant Nrf2 immunoreactivity in the spinal cord, particularly in the nucleus, compared with SNI rats treated with intrathecal DMSO (Figure 4).

**FIGURE 5:**
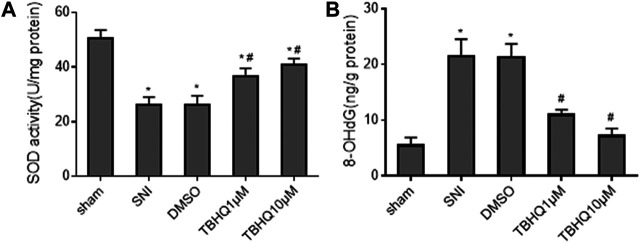
Effects of intrathecal injection of DMSO or different doses of Nrf2 agonist tBHQ on levels of antioxidant enzyme superoxide dismutase (SOD, **(A)** and oxidative stress marker 8-hydroxy-2′-deoxyguanosine (8-OHdG, **(B)** in the spinal cord of SNI rats. Sham rats without treatment served as control. Values are expressed as mean ± SE (n = 4 for each group). *p < 0.05 vs sham; ^#^ *p* < 0.05 vs SNI or DMSO.

Because Nrf2 pathway mediates oxidative stress that has been implicated in the pathogenesis of NP, we also measured the levels of antioxidant enzyme SOD and oxidative stress marker 8-OHdG in the spinal cord. Consistent with expression of Nrf2 and NQO1, the levels of SOD were markedly decreased (Figure 5A), whereas the levels of 8-OHdG (Figure 5B) were increased in SNI rats without treatment. Intrathecal tBHQ at both doses completely reversed SNI-induced changes in SOD and 8-OHdG. Of note, intrathecal DMSO did not alter SNI-induced changes in SOD and 8-OHdG.

### Time Course of Change in Expression of Sirt2 in the Spinal Cord Following SNI

**FIGURE 6:**
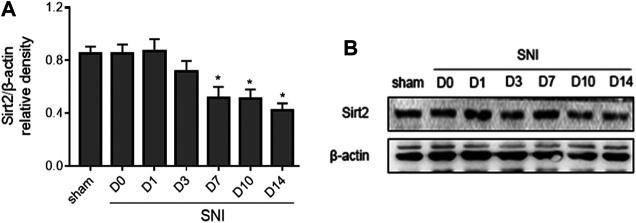
The time course of changes in expression of Sirt2 in the spinal cord in SNI rats. Sham rats served as control. Values are expressed as mean ± SE (n = 5 for each group). *p < 0.05 vs sham or baseline (day 0).

New evidence reveals that Sirt2 regulates Nrf2 pathway in different tissues. We next examined the expression of Sirt2 in the spinal cord following SNI. As shown in Figure 6, the expression of Sirt2 in SNI rats was not altered at day 1 and day 3, but it was significantly decreased from day 7 and remained lower till the end of the experiment compared to baseline or sham rats.

### Upregulation of Sirt2 in the Spinal Cord Alleviates NP, Which is Associated with Activation of Nrf2 Pathway and Decreased Oxidative Stress

**FIGURE 7:**
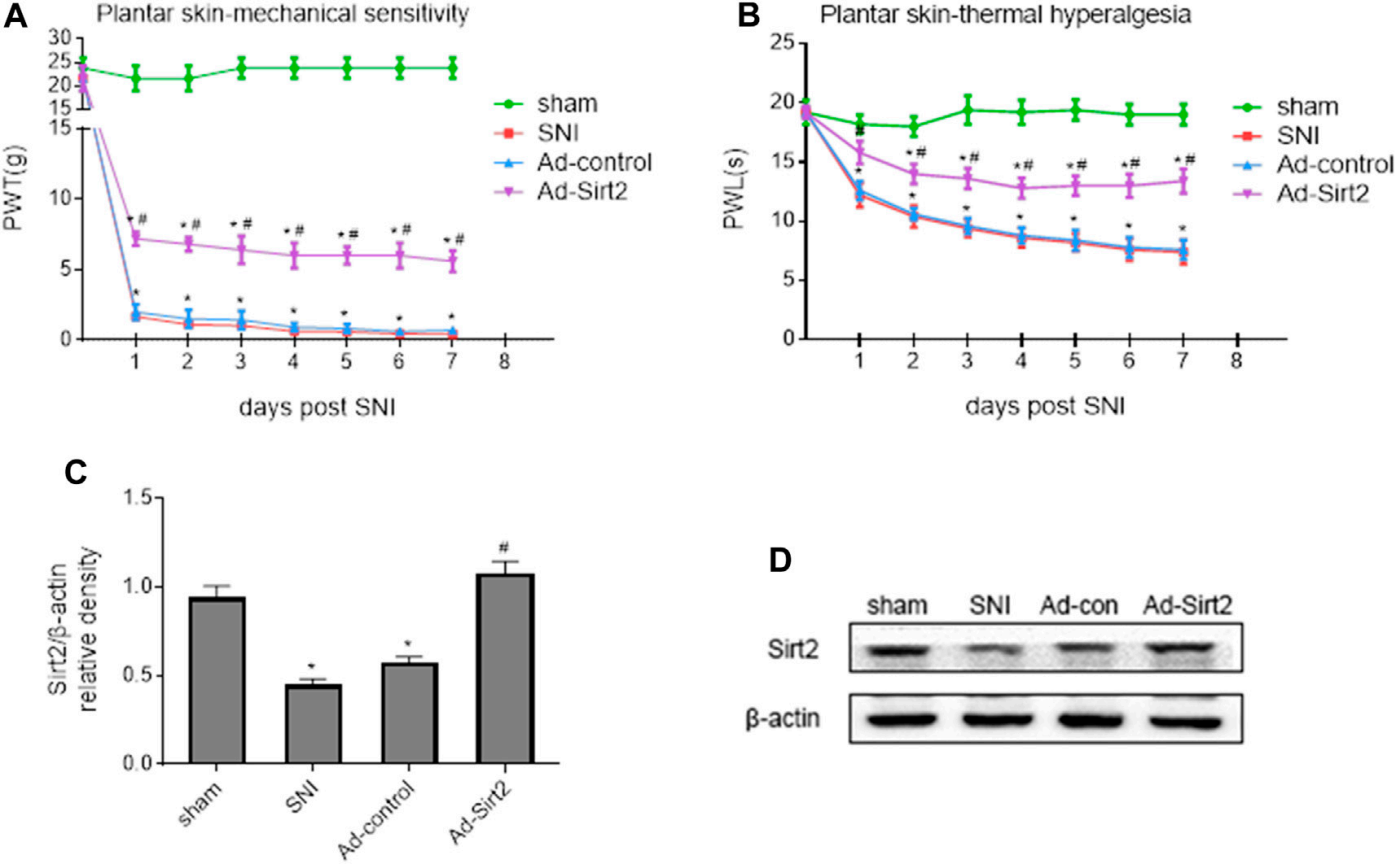
**(A,B)**: Effects of intrathecal injection of a recombinant adenovirus expressing Sirt2 and GFP (Ad-Sirt2) or control and GFP (Ad-control) on mechanical allodynia and thermal hyperalgesia in SNI rats. **(C,D)**: Effects of intrathecal injection of Ad-Sirt2 or Ad-control on expression of Sirt2 in the spinal cord of SNI rats. Sham rats without treatment served as control. Values are expressed as mean ± SE (n = 5 for each group). *p < 0.05 vs sham or baseline (day 0); ^#^ *p* < 0.05 vs SNI or Ad-control.

To further determine whether upregulation of Sirt2 in the spinal cord could alleviate NP, we treated SNI rats with intrathecal injection of a recombinant adenovirus expressing Sirt2 and GFP (Ad-Sirt2) or control and GFP (Ad-control). As presented in Figure 7, SNI rats that received intrathecal Ad-Sirt2, but not Ad-control, had significantly reduced mechanical allodynia (Figure 7A) and thermal hyperalgesia (Figure 7B) as compared to SNI rats without treatment.

The expression of Sirt2 was significantly lower in the spinal cord of SNI rats without treatment compared with sham rats. Intrathecal Ad-Sirt2 restored expression of Sirt2 in the spinal cord of SNI rats to similar level as in sham group (Figures 7C,D).

**FIGURE 8:**
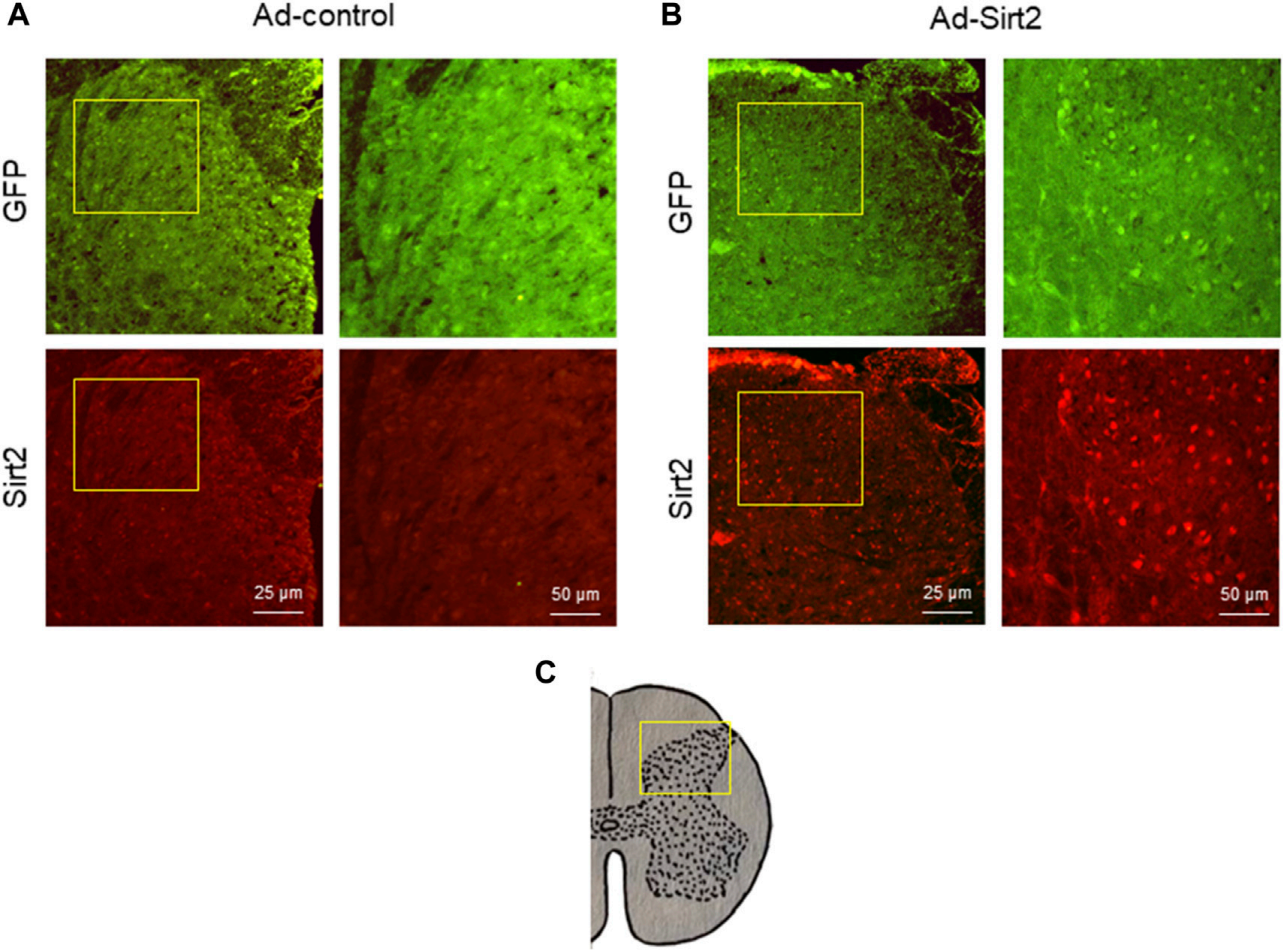
**(A,B)**: Representative confocal images showing GFP (green) and Sirt2 (red) immunoreactivity in the spinal cord of SNI rats treated with Ad-Sirt2 or Ad-control. Right column of images in Ad-control group or Ad-Sirt2 group: higher-power magnification of a section of the left images. **(C)**: Cross section of spinal cord showing where the left column of images in A and B were photographed.

Immunofluorescent study demonstrated GFP expression in the spinal cord in both SNI rats treated with intrathecal Ad-Sirt2 and SNI rats treated with intrathecal Ad-control. However, SNI rats treated with intrathecal Ad-Sirt2 had higher Sirt2 immunoreactivity than SNI rats treated with intrathecal Ad-control (Figure 8).

**FIGURE 9:**
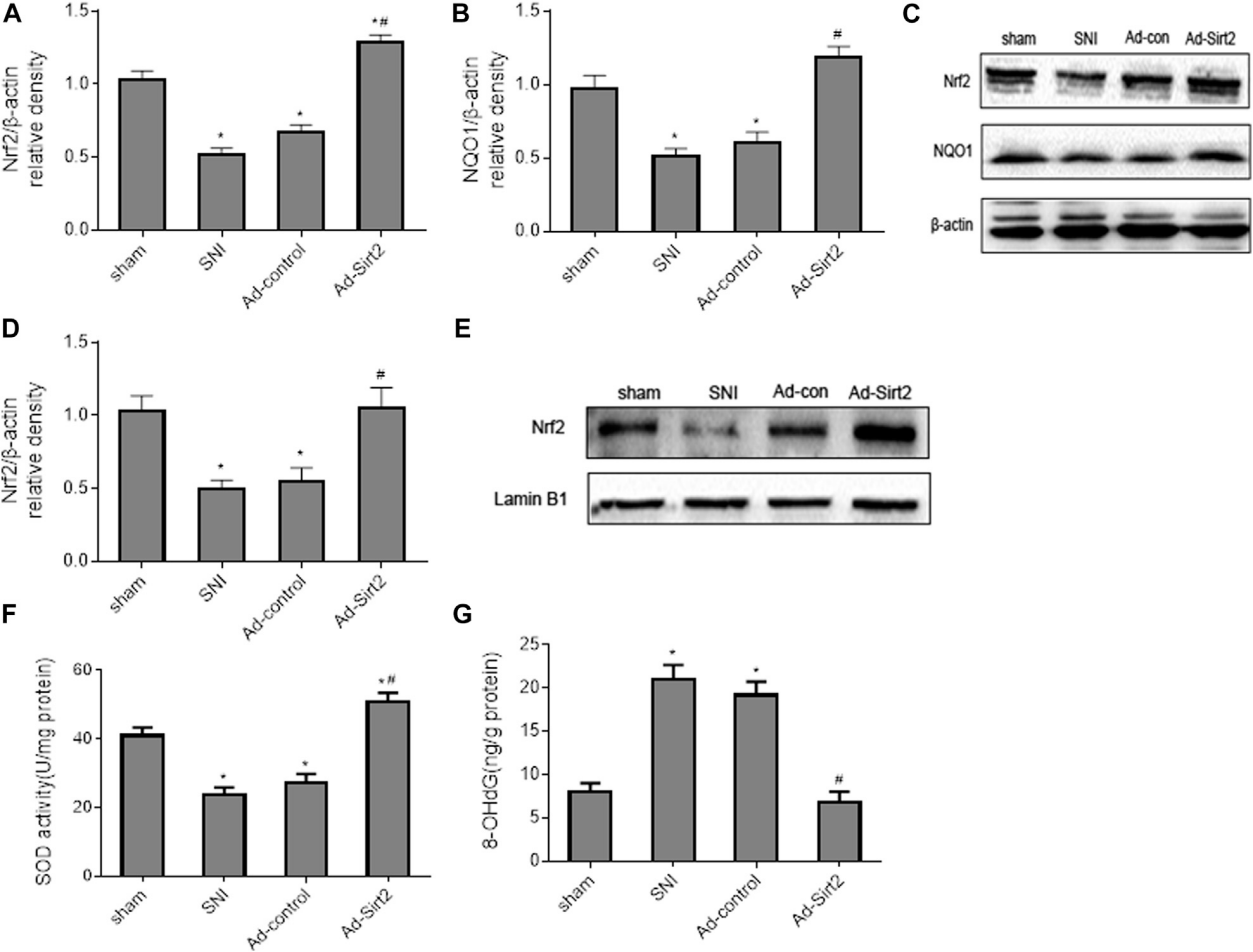
**(A–C)**: Effects of intrathecal injection of Ad-Sirt2 or Ad-control on expression of Nrf2 and its downstream target NQO1 in the whole tissue lysates of the spinal cord in SNI rats. **(D,E)**: Effects of intrathecal injection of Ad-Sirt2 or Ad-control on expression of Nrf2 in the nuclear fractions of the spinal cord in SNI rats. **(F,G)**: Effects of intrathecal injection of Ad-Sirt2 or Ad-control on levels of antioxidant enzyme superoxide dismutase (SOD) and oxidative stress marker 8-hydroxy-2′-deoxyguanosine (8-OHdG) in the spinal cord of SNI rats. Sham rats without treatment served as control. Values are expressed as mean ± SE (n = 5 for each group). *p < 0.05 vs sham; ^#^ *p* < 0.05 vs SNI or Ad-control.

Importantly, we found that expression of Nrf2 (Figures 9A,C) and NQO1 (Figures 9B,C) in the whole tissue lysates and expression of Nrf2 in the nuclear fractions (Figures 9D,E) in SNI rats treated with intrathecal Ad-Sirt2 were normalized to those observed in sham rats. Moreover, intrathecal Ad-Sirt2 restored SNI-induced changes in SOD (Figure 9F) and 8-OHdG (Figure 9G) in the spinal cord. Intrathecal Ad-control had no effect on any of these measured parameters.

## Discussion

The main findings of the present study are: 1) Nrf2 activity and its downstream target NQO1 expression in the spinal cord are downregulated in rats with SNI-induced chronic NP; 2) upregulation of Nrf2 activity restores NQO1 expression and prevents oxidative stress in the spinal cord, ameliorating mechanical allodynia and thermal hyperalgesia in rats with SNI-induced chronic NP; 3) Sirt2 expression in the spinal cord is reduced in rats with SNI-induced chronic NP; 4) overexpression of Sirt2 prevents reductions in Nrf2 activity and NQO1 expression as well as increase in oxidative stress in the spinal cord, leading to improvement of thermal hyperalgesia and mechanical allodynia.

Evidence supports that after peripheral or central nervous system injury, elevated extracellular glutamate levels activate multiple intracellular pathways including ROS formation (Carrasco et al., 2018). It is well known that increase in intracellular ROS plays a critical role in etiology of pain processes (Carrasco et al., 2018). For example, excessive ROS can promote hyperexcitability of dorsal root ganglia neurons through several mechanisms, including disrupted mitochondrial bioenergetics, which impairs energy production and ion homeostasis, resulting in spontaneous activity and degeneration of nociceptors (Grace et al., 2016). Excessive ROS may also indirectly cause neuronal hyperexcitability by promoting production of neuroinflammatory mediators (Grace et al., 2016; Ji et al., 2016). Interventions that attenuate oxidative stress have been shown to ameliorate NP in animals (Tiwari et al., 2012; Sharma et al., 2018). Nrf2 pathway plays an essential role in protecting cells and tissues from oxidative stress (Lee and Johnson, 2004; Kaspar et al., 2009; Catanzaro et al., 2017). Under physiological conditions, Nrf2 is sequestered in the cytosol by binding to its cytosolic inhibitor Keap1 and ubiquitinated for degradation. Under oxidative stress, Nrf2 dissociates from Keap1 and translocates into the nucleus, where it binds to the antioxidant response element that initiates transcription of more than 200 antioxidant-related genes, leading to upregulation of a battery of antioxidants, including SOD, glutathione peroxidase and glutathione (Lee and Johnson, 2004; Kaspar et al., 2009; Catanzaro et al., 2017). Accumulating evidence shows that Nrf2 pathway is implicated in NP. For instance, activation of Nrf2 in the spinal cord with Nrf2 activator oltipraz attenuates oxidative stress and ameliorates mechanical allodynia in a rat model of paclitaxel-induced NP (Zhou et al., 2020). Electroacupuncture increases production of antioxidants and alleviates paclitaxel-induced NP by upregulating Nrf2 activity in the dorsal root ganglion (Zhao et al., 2019). Activation of Nrf2 activity can also improve mechanical allodynia and thermal hyperalgesia in diabetes-induced NP in mice (Xu et al., 2020). In the present study, we found that expression of Nrf2 and NQO1 in the whole tissue lysates of the spinal cord in SNI rats tended to be higher at day 1, but gradually decreased from day 7 following SNI. Expression of Nrf2 in the nuclear fractions in SNI group was significantly increased at day 1, but then start to gradually decrease. These results suggest that expression of Nrf2 and translocation of Nrf2 into the nucleus are increased to protect cells from SNI-induced oxidative stress and cellular damage in the early stages of NP. However, both expression of Nrf2 and translocation of Nrf2 into the nucleus are downregulated over time in rats following SNI, leading to increased mechanical allodynia and thermal hyperalgesia. This finding is consistent with a recent study showing decreased Nrf2 expression in the spinal cord in rats following SNI. Our data also demonstrated that activation of Nrf2 pathway in the spinal cord with Nrf2 agonist tBHQ ameliorated NP, which was accompanied by increased Nrf2 downstream target NQO1 and antioxidant enzyme SOD and decreased oxidative stress marker 8-OHdG. These observations indicate that activation of Nrf2 pathway in the spinal cord is downregulated in rats following SNI, which enhances vulnerability of spinal cord tissues to oxidative insults and leads to increased oxidative stress, contributing to the development of chronic NP.

The sirtuins are a class of evolutionarily highly conserved nicotinamide adenine dinucleotide (NAD+)-dependent histone deacetylases that play important roles in many cellular biological processes (Singh et al., 2018; Zhang et al., 2020). The sirtuin family of proteins consists of seven members (Sirt 1–7) (Singh et al., 2018; Zhang et al., 2020) and Sirt2 is an important member of the sirtuin family that plays a vital role in regulating oxidative stress, inflammation, and mitochondrial function (Zhang et al., 2020). Previous study reported that Sirt2 deacetylated FOXO3a in response to oxidative stress and reduced ROS production by upregulating expression of FOXO3a target genes in NIH3T3 cells (Wang et al., 2007). Additionally, Sirt2 has been shown to inhibit mitochondrial autophagy and protect annulus fibrosus cells from oxidative stress-induced apoptosis via regulation of peroxisome proliferator-activated receptor gamma coactivator 1-alpha (Xu et al., 2019). A recent study reported that NADH-induced increase in the expression of nuclear Nrf2 in PC12 cells was prevented by both Sirt2 siRNA and Sirt2 inhibitor; Sirt2 siRNA also blocked the NADH-induced increases in the levels of intracellular antioxidant glutathione (Cao et al., 2016). This observation suggests that activation of Nrf2 can be regulated by Sirt2. Sirt2 is widely distributed in the peripheral and central nerve system tissues, including microglia (Wang et al., 2016), dopaminergic neurons (Szego et al., 2017), hippocampal neurons, spinal cord (Park et al., 2016) and dorsal root ganglion (Zhang and Chi, 2018). To explore the mechanism that regulates Nrf2 pathway in the development of chronic NP, we measured the expression of Sirt2 in the spinal cord. Our results showed that Sirt2 expression in the spinal cord was gradually decreased in rats following SNI. More importantly, we found that overexpression of Sirt2 in the spinal cord of SNI rats restored Nrf2 activity and NQO1 expression, reversed SNI-induced changes in levels of antioxidant enzyme SOD and oxidative stress marker 8-OHdG, and significantly improved thermal hyperalgesia and mechanical allodynia. These findings indicate that reduction in Nrf2 activity and NQO1 expression and increase in oxidative stress in the spinal cord in rats following SNI are mediated by Sirt2, which plays an important role in the development of chronic NP.

One limitation of this study should be acknowledged. The SNI animal model has been reported to exhibit persistent (>6 months) NP (Decosterd and Woolf, 2000). However, our current study only focused on the role of Nrf2 activity in the spinal cord in regulating NP at an early stage. Further studies are necessary to examine whether Nrf2 activity in the spinal cord also plays an important role in mediating NP at a later stage.

In conclusion, the present study demonstrates that peripheral nerve injury downregulates Sirt2 expression in the spinal cord, which inhibits Nrf2 activity, leading to increased oxidative stress and the development of chronic NP. Current medications used for neuropathic pain offer only symptomatic relief without treating the underlying pathophysiology. In addition, these medications are associated with various dose-limiting side effects (Fernandes et al., 2018, Bidve et al., 2020). A better understanding of cellular and molecular mechanisms of NP may provide the key to development of effective NP therapies. That have minimal side effects. This study provides new evidence that Nrf2 pathway in the spinal cord plays an important role in regulating chronic NP. Systemic or intrathecal delivery of Nrf2 activators to increase spinal Nrf2 levels or administration of small-molecule Nrf2-enhancing compounds to induce endogenous Nrf2 activity and expression in the spinal cord, may be therapeutic approaches for treatment of patients with chronic NP.

## Data Availability

The original contributions presented in the study are included in the article/Supplementary material, further inquiries can be directed to the corresponding author.
